# Binding of RbFox proteins at weak 5’splice site of A2 induces its alternative splicing in non-muscle myosin heavy-chain IIA mRNA

**DOI:** 10.1016/j.jbc.2026.111443

**Published:** 2026-04-09

**Authors:** Ditipriya Mallick, Nitish Pal, Sampurna Dutta, Siddhartha Sankar Jana

**Affiliations:** School of Biological Sciences, Indian Association for the Cultivation of Science, Kolkata, India

**Keywords:** alternative splicing, non-muscle myosin II, neuronal splice factors, RbFox isoforms

## Abstract

The brain-specific alternative splicing of MYH9 pre-mRNA causes insertion of a 63-nucleotide exon (21-amino acid, A2) at the loop2 region of motor domain in non-muscle myosin heavy chain IIA (NMHCIIA), generating an inactive mechanoenzyme, NMIIA2. But the mechanism of such a splicing event in a neuronal cell is yet to be explored. Here, we report that the skipping of the A2 exon in non-neuronal cells arises due to the presence of a weak 5′ splice site, as increasing the strength of 5′ splice site prevents such skipping. The binding of neuron-specific splice factors, RbFox (s), at the 5′ splice site overcomes the effect of a weak 5′ splice site, leading to the expression of NMIIA2 protein in the neuronal cells. Our findings suggest that the presence of a weak 5′ splice site and its interaction with RbFox specify alternative splicing of A2 exon in the neuronal cells.

Alternative splicing is a key regulatory mechanism in pre-mRNAs that governs the differential expression of spliced isoforms in a cell-type-specific manner across various developmental stages. Brain tissue exhibits the highest percentage of alternative splicing, and displays more than 400 different alternative splicing events (including cassette exon, alternative donor, alternative acceptor, retained intron, *etc.*) by which it can generate proteins with potential roles in neuronal maturation, synapse formation and overall brain function (1, 2, 3).

Brain-specific alternative splicing has recently been reported in non-muscle myosin II (NMII), and mutations in these spliced isoforms lead to autosomal dominant hearing loss (DFNA4) in humans (4). NMII consists of three pairs of polypeptides: two heavy chains (HC) that dimerize through the tail domain and hydrolyze ATP through the head domain to pull actin filaments, two regulatory light chains (RLCs) that regulate NM II activity through phosphorylation and two essential light chains (ELCs) that stabilize the HC. HC is encoded by three different genes, *MYH9*, *MYH10* and *MYH14* encoding three paralogs NMHC IIA, -IIB and -IIC, respectively, in mammalian cells (5, 6, 7). The pre-mRNAs of NMHC IIB and -IIC genes can undergo tissue-specific alternative splicing at loop1, near the ATP-binding region and loop2, near the actin-binding region, of motor head domains. Alternative splicing at loop2 results in the inclusion or skipping of a 63-nucleotide (nt) exon in the NMHC IIB gene and a 99-nt exon in NMHC IIC gene, which generate the NMHC IIB0/IIB2 and NMHC IIC0/IIC2 isoforms, respectively (7, 8). Recently, Das *et al.*, 2023, reported that alternative splicing of a 63-nt exon at loop2 region of NMHC IIA (A2 exon) leads to brain-specific expression of NMIIA2 isoform. Inclusion of the A2 exon in NMIIA2 alters the mechano-enzymatic properties of NMIIA, as NMIIA2 protein lacks measurable actin-gliding activity and shows low actin-activated ATPase activity *in vitro* (9). Hence, elucidating the mechanism of alternative splicing at loop2 in NMHC IIA gene is warranted to understand the brain-specific expression of NMIIA2 and to highlight the overall activity of the constitutively expressed major isoform, NMIIA during circadian rhythm and in various neurodegenerative diseases.

In this study, we demonstrate that the alternative splicing at loop2 region of NMHC IIA mRNA causes skipping of the A2 exon in non-neuronal tissues due to the presence of a weak 5′ splice donor site. However, the binding of neuron-specific splice factors, RbFox proteins, to the weak 5′ splice site allows exon recognition and promotes the brain-specific inclusion of the A2 exon in NMHC IIA mRNA. Altogether, the alternative splicing of NMHC IIA is governed by the presence of weak splice sites and brain-specific RbFox splice factor.

## Results

### The genomic location of the alternative A2 exon in the NMHC IIA gene and its conservation across species

**Figure 1:**
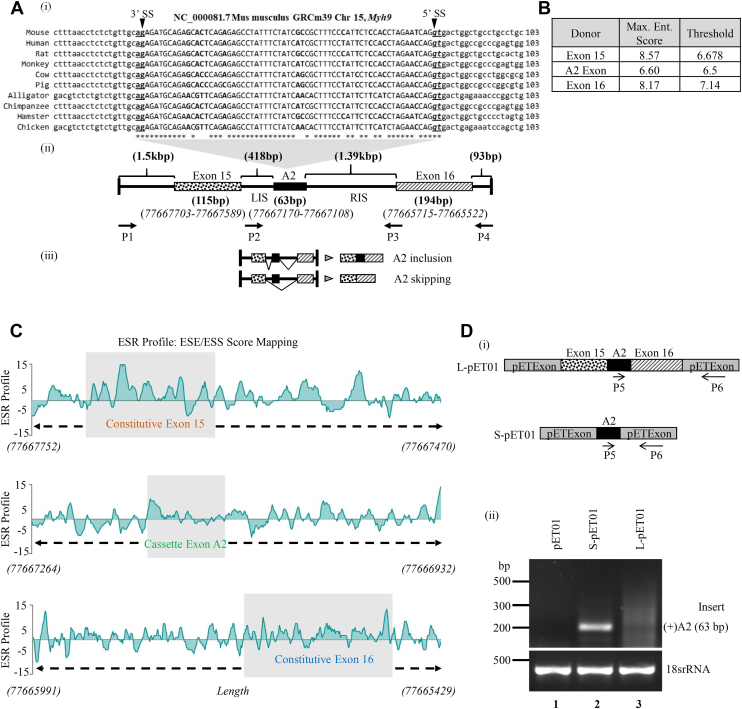
**Alternative splicing of A2 exon in S-pET01 and L-pET01 and analysis of its cis-regulatory sequences.***A*, (i), the genomic location of mouse NMHC IIA gene, covering A2 exon (uppercase letters) and the flanking introns (lowercase letters). GenBank accession # NC_000081.7. The boldface, underlined letters denote the 3′ splice site and the boldface, italics and underlined letters denote the 5′ splice site of the A2 exon. Sequence alignment of the genomic region across the 10 species. *Asterisks* (∗) indicate sequence identity and boldface letters denote non-identity. (ii) A schematic diagram depicting the A2 cassette exon, its flanking introns (LIS and RIS), and the constitutively expressed exons 15 and 16 and their flanking introns. *Arrows* indicate the positions of P1-P4 and P2-P3 primer pairs used in L-pET01 and S-pET01 cloning, respectively. The respective genomic position of each of the exons is given in brackets underneath the respective exons (below). (iii) A schematic diagram showing the alternative splicing events and the transcripts. *B*, the MaxEntScan score of the 5′ splice site of the exons A2, 15 and 16. *C*, enhancer to silencer ratio (ESR) profile of the exons 15, A2 and 16, mapped from HSF 3.0. *D*, a schematic map of the A2 transcripts as generated from S-pET01 and L-pET01, arrows indicate the primers used for RT-PCR (i). RT-PCR analysis using A2-specific primers of spliced products generated from S-pET01 and L-pET01 transfected Neuro2A cells (ii).

Previously, Das *et al.* 2023 showed that NMHC IIA exhibited an alternatively spliced A2 insert at loop2 region of the motor head domain in brain tissue (9). The A2 exon of 63 nt is located between exon 15 and 16, and its tissue-dependent expression suggests that splicing at this region occurs either by cassette-type exon skipping or exon inclusion. Moreover, the sequence alignment of the genomic region corresponding to the A2, B2 or C2 exons showed that the 3′ acceptor site (*ag*) and the 5′ splice donor site (*gt*) (Fig. 1*A* (i), S1A-B, bold, underlined (*ag*) and italicized (*gt*)) of each of the loop2 inserts were conserved. The sequence identity of A2 exon across the species was 79.63%, whereas that of B2 and C2 exons was 85.7% and 58.5%, respectively, suggesting the importance of A2 exon-encoding amino acids in NMII functionality and structural conformation (Fig. S1, *A* and *B*). To understand why the A2 exon is predominantly skipped in the majority of tissues, we analyzed the strength of the splice donor and acceptor sites of the exon (Figs. 1*B* and S1*C*(i)). The strength of the 3′and 5′ splice sites was determined by their homology to the consensus sequence complementary to the RNA Recognition Motifs (RRM) of snRNA in U2 and U1 snRNP, respectively (10, 11, 12). We found that the strength of the 5’-splice site of A2 exon was 6.60, which was suboptimal, as the mean score of an exon containing constitutive 5′ splice donor site was above 8.0, as in exons 15 (8.57) and 16 (8.17) (Fig. 1*B*). In contrast, the 3’splice site of A2 exon had optimal strength (9.02) (Fig. S1*C*(i)), suggesting that the weak 5′ splice site can play an important role in its skipping by reducing the recognition of the exon-intron boundary by the spliceosome complex.

The exon recognition is also regulated by cis-regulatory sequences, exonic splicing enhancers (ESE) and exonic splicing silencers (ESS), which facilitate exon inclusion and skipping, respectively. A high-scoring exon sequence ratio (ESR), ESE/ESS, in a sequence determines exon recognition. The ESR profile of the genomic region consisting of exons 15, 16, and A2 showed that exons 15 and 16 had higher mean score of ESR (≥5) while A2 exon sequence had a low ESR score, suggesting that A2 exon has a lower abundance of ESE motifs and reduced affinity for ESE-binding proteins compared to the constitutive exons 15 and 16 (Fig. 1*C*). To validate the *in silico* predictions of the cis-regulatory sequence elements, we cloned the mouse genomic region that consists of A2 exon its flanking introns and the constitutively expressed exons 15 and 16 of *Myh9* gene, into the pET01 ExonTrap Cloning Vector designed as reporter for minigene splicing assays. Two genomic fragments were cloned: the 3.8-kb-long fragment (L-pET01) consists of the exon 15, A2 and 16 and their flanking introns while the 1.8 kb short fragment (S-pET01) consists of only the A2 exon and its flanking introns (Left Intronic Sequence, LIS and Right Intronic Sequence, RIS) (Fig.1*A* (ii-iii)). Therefore, transcripts from L-pET01 included exon 15, A2, exon16 while those from S-pET01, included only the A2 exon (Fig. 1*D* (i)). Interestingly, pET01-transfected Neuro2A cells produced a 310 bp band, consistent with efficient transcript splicing of pET01 (Fig. S1*D* (ii)). RT-PCR analysis showed that A2 transcripts were detected in Neuro2A cells transfected with S-pET01 (*lane 2*) but not with either pET01 (*lane1*) or L-pET01 (*lane3*) (Figs. 1*D*(ii) and S1*D*(ii)), suggesting that the exogenous expression of A2 exon is minimal in Neuro 2A and the distal introns flanking exon 15 and 16 may have an inhibitory effect on A2 inclusion.

### The 5’splice site of A2 exon regulates its alternative splicing

**Figure 2:**
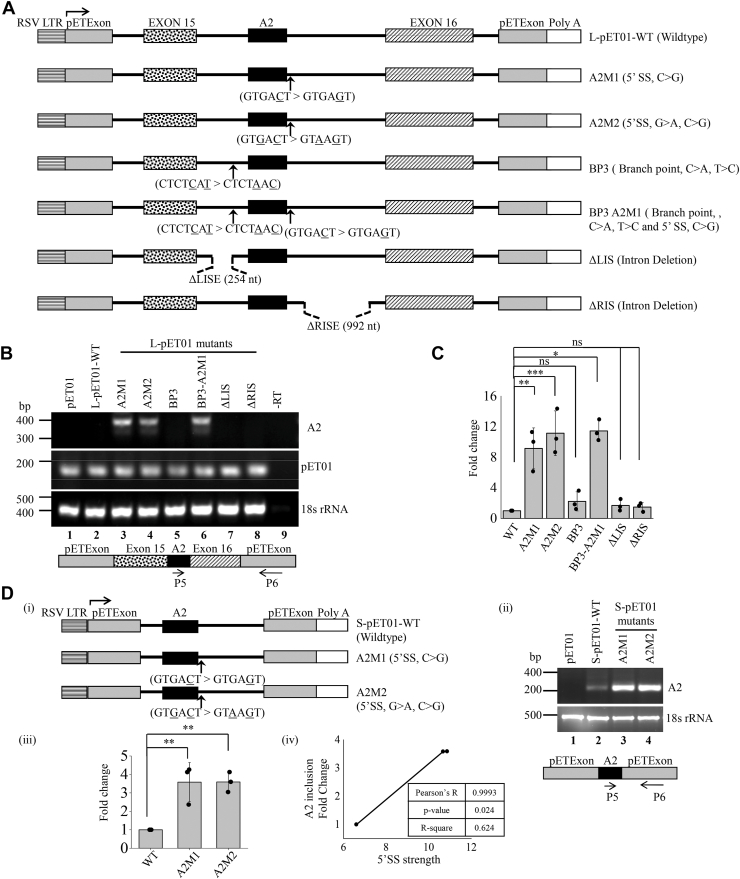
**Increasing the strength of 5′ splice donor site causes A2 exon inclusion in transcripts generated from L-pET01 and S-pET01 minigenes.***A*, a schematic map of the genomic region cloned into L-pET01. The mutation positions (*arrows*) and the mutated nt (underlined) depicted for A2M1, A2M2, BP3, and BP3-A2M1. The broken lines in ΔLIS and ΔRIS denote the deletion region. *B*, RT-PCR analysis using A2-specific primers showing spliced product generated from L-pET01 WT (wildtype) and MT (mutants) transfected in Neuro2A cells. PCR product of only pET01 exons and 18 s rRNA were used as transfection control and loading control, respectively. A schematic map of the A2 transcripts from L-pET01, *arrows* indicate the positions of the primers (below). *C*, expression fold change of A2 transcripts in L-pET01 WT and MT. *D*, a schematic map of the genomic region cloned into S-pET01 (i) and RT-PCR analysis using A2-specific primers of spliced products generated from S-pET01 WT and MT in Neuro2A cells. 18 s rRNA was used as a loading control. A schematic map of the A2 transcripts from S-pET01, arrows indicate the positions of the primers (below) **(**ii). Fold change of expression of A2 transcripts in S-pET01 WT and MT (iii). Pearson’s correlation (R) scatterplot of A2 inclusion fold change and 5′SS strength (iv). ∗, *p* < 0.05; ∗∗, *p* < 0.01; ∗∗∗, *p* < 0.001; ns, non-significant; WT vs A2M1, A2M2, BP3, BP3-A2M1, ΔLIS or ΔRIS.

The previous data indicated that the presence of weak 5′ splice site at A2 exon may repress A2 recognition during pre-mRNA splicing. To assess the involvement of cis-regulatory sequences in the splicing of A2 exon, we generated mutations at the 5′ splice site, branch point sequences, and flanking intronic sequences in L-pET01 and S-pET01, and checked for A2 exon skipping or inclusion (Fig. 2*A*). First, using MaxEntScan algorithm, we noticed that a single nt substitution at the 5′ splice site (C>G, A2M1) increased the strength of the 5′ splice site from 6.60 to 10.67, while a substitution at another position (G>A, A2M2) further increased the strength to 10.86 (Fig. S2*A*(i)) (11). Interestingly, both A2M1 and A2M2 in L-pET01 and S-pET01 backbones showed an increase in A2 transcripts (9.16 ± 2.56, L-pET01; 3.58 ± 0.49, S-pET01 for A2M1 and 11.15 ± 0.41, L-pET01; 3.59 ± 0.87, S-pET01 for A2M2) as compared to wildtype (Fig. 2, *B*–*D*), confirming that A2 exon inclusion depends on the strength of 5’splice site. Second, using HSF3.0 we considered a regulatory branch-point sequence located 54 bp upstream of the A2 exon, which has a consensus value of 92.5 (Fig. S2*A*(ii)) (13). Notably, although the substitution of two nucleotides (C>A and T>C, BP3) at the branch point reduced the consensus value to 84.5, it showed the decreased abundance of A2 exon same as wildtype (Fig. 2*B, lane 2 vs 5*). A2 transcripts were detectable only if the strength of 5′ splice site was increased in BP mutants (Fig. 2*B, lane 6*, BP3-A2M1). Third, we deleted a region of LIS or RIS in L-pET01, where intronic splicing enhancer or silencer (ISS or ISE) sequence motifs were enriched to assess the effect of the flanking intron sequence on the A2 exon inclusion (Fig.1*A* (ii)). ISS motifs have a prominent effect in overcoming the effect of ISE motifs on exon inclusion (14). We noticed that both ΔLIS and ΔRIS did not increase the abundance of A2 transcripts in Neuro2A cells (Fig. 2, *B* and *C*) suggesting that the ISS motifs in the LIS or RIS may not regulate A2 exon inclusion. Altogether, these findings suggest that A2 exon inclusion is regulated only by the strength of A2 exon 5’splice site, but not by the ISS motifs in the LIS or RIS, or by the branch point sequence.

**Figure 3:**
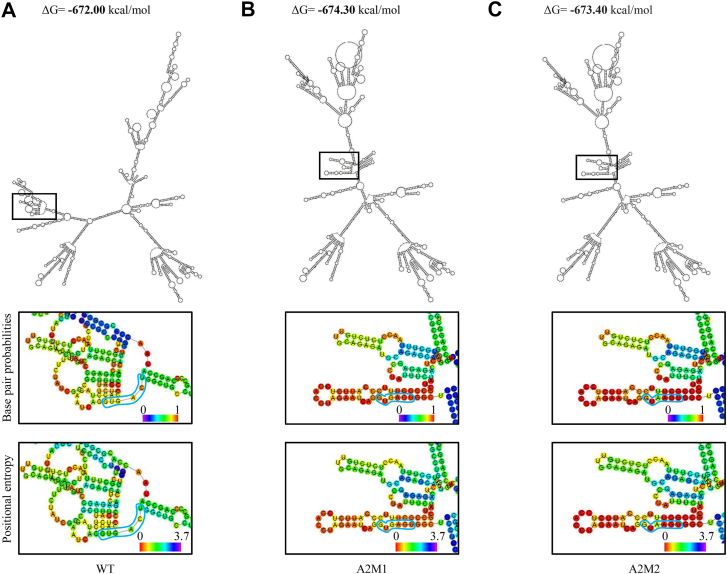
**RNA secondary structures of A2M1 and A2M2 show increased accessibility of 5′ splice donor site.***A–C*, RNA secondary structures generated using RNAfold webserver of the region corresponding to the A2 exon and flanking intronic sequences in WT (A) or MT (A2M1 and A2M2). *B* and *C*, the boxed region comprising of the stem-loop structure of A2 exon is enlarged (below) to show the base pair probabilities and positional entropy of the nucleotides in the RNA model, where the nucleotides at 5′ splice sites of WT and MT are outlined in *blue*.

Since the cis-regulatory sequences regulate splicing either through the nucleotide sequence or pre-RNA secondary structure, or both, we investigated the role of the RNA secondary structure, specifically, at the 5′ splice donor site of A2 exon. Using the RNAfold webserver, we found that the Minimum Entropy Structures that consists of the A2 exon and the flanking intronic sequences do not significantly differ in the stability (ΔG, WT: −672 kcal/mol, A2M1: −674.30 kcal/mol, A2M2: −673.40 kcal/mol) (15). However, the A2M1 and A2M2 mutants show increased base pairing probabilities and decreased positional entropy, indicating that mutations at the 5′ SS increased local structural stability (Fig. 3, enlarged). This conformation may increase the accessibility of the splice factors to bind to the A2 exon. Therefore, these data indicate that both the strength and accessibility of the 5′ splice site determine A2 exon inclusion (Fig. 2*D* (iv)). To understand if this mechanism of A2 exon splicing is conserved in human, we performed an analysis of the 5′SS and 3′SS strengths and the RNA secondary structures using the human genomic DNA region corresponding to the A2 exon and its flanking introns. We found that both the strength and local RNA structural stability of the 5′ SS were higher in the mutants compared with the wildtype, which indicates that humans and mice share a similar regulation of A2 exon splicing (Fig. S2, *B* and *C*).

### RbFox splice factors promote the splicing of A2 exon

**Figure 4:**
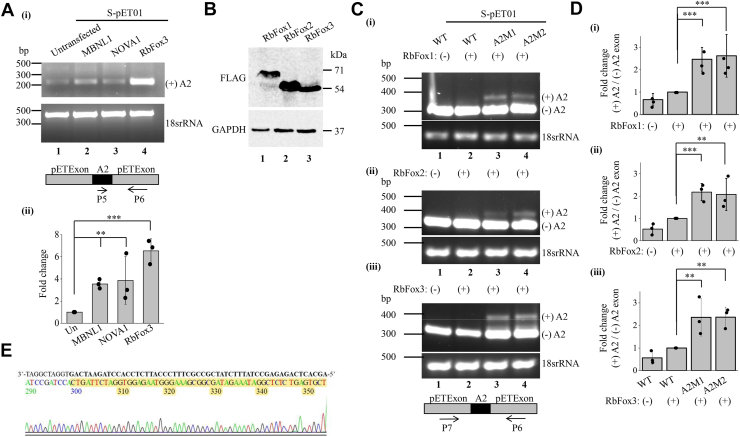
**RbFox proteins bind to the 5′ splice site to promote A2 exon inclusion in transcripts generated from S-pET01 minigene.***A*, (i) RT-PCR analysis of spliced products generated from S-pET01 WT co-transfected with or without MBNL1, NOVA1 or RbFox3 in Neuro2A cells. 18 s rRNA was used as a loading control. A schematic map of A2 transcript and arrows indicate the positions of the primers (below). (ii) Fold change of expression of A2 transcripts. *B*, immunoblot showing the expression of RbFox1, 2 and 3-FLAG in Neuro2A cells. GAPDH used as a loading control. *C*, RT-PCR analysis using pET01 exon-specific primer of spliced products generated from S-pET01 WT or MT co-transfected with or without RbFox1 (i), RbFox2 (ii) or RbFox3-FLAG (iii), in Neuro2A cells. 18 s rRNA was used as a loading control. A schematic map of the transcripts and arrows indicate the positions of the primers (below). *D*, fold change of expression of A2 transcripts in cells co-transfected with S-pET01 WT or MT, and RbFox1 (i), RbFox2 (ii) or RbFox3-FLAG (iii). *E*, representative chromatogram showing inclusion of the A2 exon (highlighted in *yellow*) in the 373 bp A2 transcript generated from A2M2-S-pET01, in presence of RbFox3. ∗∗, *p* < 0.01; ∗∗∗, *p* < 0.001; WT (+) vs A2M1 (+) or A2M2 (+).

The brain-specific inclusion of weak 5′ splice site containing A2 exon in NMHC IIA transcript and its skipping in other tissues suggests the interaction of brain-specific splice factors with the cis-regulatory elements. We used the SpliceAid2 to detect the putative neuronal splice factors that may bind to the region corresponding to the A2 exon. SpliceAid2 detected an array of similar splice factors binding across the A2 exon and A2 exon-intron boundary sequences in both mice and humans (Table S1 and Fig. S2*D*) (16). In mice, we selected the splice factors based on their binding to the 5′ SS, such as RbFox3 (binds to 5′SS), NOVA1 and MBNL-1 (bind to non-5′SS), and co-transfected each of them with WT or A2M2-S-pET01 in Neuro2A cells to assess A2 splicing. We found that MBNL1 and NOVA1 caused significant increase in A2 exon inclusion, although not to the same extent as RbFox3 (∼3.8-fold for MBNL1 and NOVA1, ∼6.5-fold for RbFox3 in wildtype S-pET01), albeit with more A2 exon splicing in A2M2-S-pET01 (Figs. 4*A* and S3, *D*–*G*). The distribution profile of expression of RbFox splice factors in the Human Protein Atlas showed that RbFox3 expression was restricted to post-mitotic neuronal cells, unlike its other isoforms RbFox1 and 2 (17). So, we assessed if RbFox1 and 2 could regulate A2 exon inclusion in neuronal cells. RbFox3 increased A2 inclusion by ∼3 and 4-fold whereas both RbFox1 and 2 showed ∼1.1 and 3-fold inclusion as compared to RbFox untransfected cells but transfected with wild-type L-pET01 and S-pET01, respectively, suggesting that RbFox3 maybe the predominant splice factor responsible for A2 inclusion in neuronal cells (Figs. 4*B* and S3, *B* and *C*). Of note, the inclusion was further increased in the presence of A2M1 and A2M2 by RbFox(s) as A2 transcript was significantly detectable using primers flanking A2 exon (Fig. 4, *C* and *D*), which we confirmed through sequencing of the upper band ((+)A2) excised from the RT-PCR gel of A2M2 and RbFox3 co-transfected Neuro2A cells (Fig. 4*E*).

**Figure 5:**
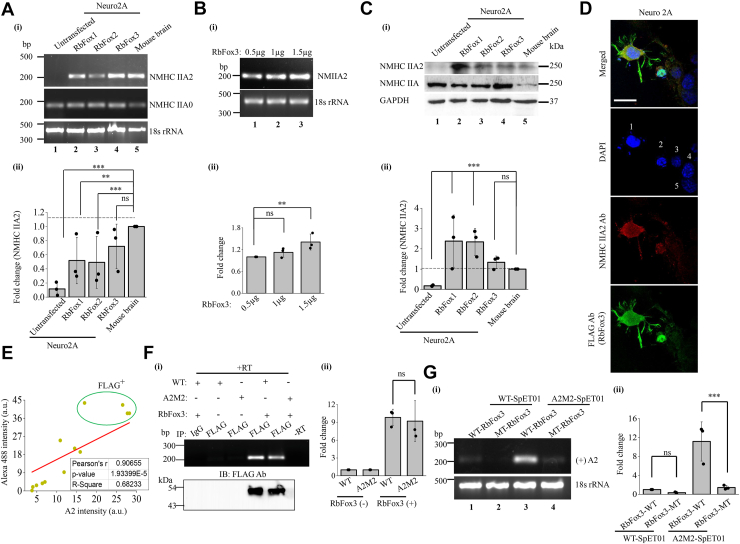
**Exogenous RbFox(s) induce the endogenous expression of NMIIA2.***A*, detection of endogenous A2 and A0 transcripts by RT-PCR analysis of Neuro2A cells un-transfected or transfected with FLAG-RbFox1, 2 or 3. 18 s rRNA was used as a loading control. (i) and quantification of fold change of endogenous A2 transcripts. Fold change was calculated by considering relative intensity of mouse brain tissue lysate as “1” (ii). *B*, RT-PCR using A2 exon-specific primers to detect the expression of endogenous A2 transcripts in Neuro2A cells transfected with different amounts of RbFox3-FLAG (0.5–1.5 μg) (i) and quantification of fold change (ii). *C*, representative immunoblots of NMHC IIA2, NMHC IIA and GAPDH from Neuro2A cell lysates transfected with or without RbFox1,2 or 3. (i) and quantification of expression fold change of endogenous NMHC IIA2, calculated by considering relative intensity of mouse brain tissue lysate as “1” (ii). *D*, representative confocal images of Neuro2A cells transfected with RbFox3-FLAG, stained with NMIIA2, FLAG antibodies and counterstained with DAPI, showing FLAG-positive (cells 1 and 2) and negative cells (cells 3–5). *E*, Pearson’s correlation scatterplot (R) of Alexa 488 intensity indicating FLAG^+^cells and A2 intensity. *F*, detection of A2 transcripts using A2 exon-specific primer in RT-PCR analysis and RbFox3 protein in representative immunoblot probed with FLAG antibody using RIP pellets from Neuro2A cells co-transfected with or without RbFox3 and WT or A2M2-S-pET01. IgG was used as negative control (i) and quantification of fold change of A2 transcripts (ii). *G*, RT-PCR analysis using A2 exon-specific primer of spliced products generated from S-pET01 wildtype or mutant (A2M2) co-transfected with wildtype (WT) or mutant (MT) RbFox3-FLAG, in Neuro2A cells. 18 s rRNA was used as a loading control (i) and quantification of fold change of A2 transcripts (ii). ∗∗, *p* < 0.01; ∗∗∗, *p* < 0.001; ns, non-significant; Brain vs untransfected, RbFox1, RbFox2 or RbFox3, 0.5 μg vs 1.0, 1.5 μg RbFox3. Scale bar: 20 μm (*D*).

We also checked for the endogenous expression of A2 transcripts, NMHC IIA2 mRNA, in presence of exogenous RbFox1, 2, and 3, and found that each of them could induce expression of endogenous NMHC IIA2 mRNA in Neuro2A cells (Fig. 5*A*). The inclusion of A2 exon was increased by 1.4-fold upon increasing the amount of exogenous RbFox3 (Fig. 5*B*). Additionally, the transient expression of RbFox3 could also induce the endogenous expression of NMHC IIA2 at the translational level (Fig. 5, *C*–*E*). Therefore, the expression of NMHC IIA2 was observed both at RNA and protein levels in cells in presence of exogenous RbFox. These data support that lower abundance of RbFox3 in non-neuronal cells along with the presence of weak 5′ splice site (or the absence of interactions between splice factors and the 5′ splice site) may promote A2 exon skipping.

### Binding of RbFox splice factors to the 5’splice site of A2 exon

To assess if RbFox3 protein binds to the 5′ splice site of A2 exon in the pre-mRNA, we immunoprecipitated FLAG-tagged RbFox3 and found that both the wild-type A2 transcript containing weak 5′ splice site and transcript containing 5′ splice site mutation (A2M2) were detected in the RbFox3 immunoprecipitate, suggesting that exogenous RbFox3 may bind to the 5′ splice site of A2 exon (Fig. 5*F*). To further investigate if the RRM of RbFox3 determines the recognition of the 5’splice site of A2 exon to regulate its splicing, we mutated the active residue, F142 in RbFox3 RRM to alanine (F142A), which can disrupt the binding of RbFox3 to target RNAs (18, 19). We co-transfected RbFox3-WT or RbFox3-F142A with WT-S-pET01 or A2M2-S-pET01 and assessed A2 inclusion. Indeed, we found that RbFox3-WT could increase the expression of A2 exon by 11-fold in A2M2-S-pET01, while the RbFox3-F142A mutant (RbFox3-MT) reduced A2 transcript expression to 4-fold as compared to the wildtype-S-pET01 (Fig. 5*G*). Altogether, our findings suggest that both factors: the availability of the 5′ splice site of A2 exon and a functional RRM of RbFox3, are necessary for the inclusion of A2 exon.

## Discussion

In this study, we demonstrate a mechanism of alternative splicing of mouse NMHC IIA pre-mRNA, Myh9, which shows retention of a 63-nt A2 exon in neuronal cells and skipping in non-neuronal cells. The size of exons and introns, the strengths of 5′ splice donor and 3′ acceptor sites, the location and sequence of branch point, poly-pyrimidine tracts, and exonic splicing regulators (such as exonic/intronic enhancers or silencers), are the cis-acting regulatory factors in the pre-mRNA that allow the recognition of alternative exons through proper binding of snRNPs during splicing. Additionally, the expression of trans-regulatory factors like RbFox, NOVA, MBNL, nPTB, and SRRM4 splice factors can promote or repress the binding of the snRNPs through cooperative binding to the pre-mRNA through their RRMs which determine the brain-specific expression of the spliced isoforms (20, 21, 22, 23, 24, 25). Particularly, the RbFox family proteins such as RbFox1, 2 and 3, are crucial splice factors in neurodevelopment, as RbFox depletion in neurons causes misregulation of more than 500 alternatively spliced cassette exons and their binding to the 3′ or 5′ splice sites can cause exon skipping or inclusion, respectively. RbFox3 has been shown to promote neuronal differentiation by driving alternative splicing of Numb, a signaling adaptor protein, by binding to an upstream intronic UGCAUG element and promoting exon skipping; loss of RbFox3 leads to inclusion of this exon and impairs neuronal differentiation *in vivo*. Importantly, dysregulation of RbFox-dependent splicing has been implicated in neurodevelopmental disorders, including autism, where reduced RbFox1 expression correlates with aberrant splicing of synaptogenesis-related genes. Collectively, these studies highlight overlapping yet distinct roles of RbFox family members in orchestrating developmental stage- and tissue-specific splicing programs during neural development, in coordination with other splicing regulators such as PTBP, NOVA, and MBNL, which together control early and late splicing transitions during neuronal maturation (1, 24, 26, 27).

We provide evidence that variability in the strength of 5′ splice donor site may be another key regulatory mechanism that facilitates splice site selection, and alternative splicing (28). Brain tissue might have a prevalence of 5′ splice sites of suboptimal strength with low sequence similarity to the consensus sequence, which enables the abundant alternative splicing observed for several genes expressed in the brain. Similar to NMIIA, both NMIIB and –IIC paralogs show brain-specific alternative splicing at the loop2 region. However, unlike the A2 exon, which has a weak 5′ splice donor site, both B2 and C2 exons of NMIIB2 and –IIC2 have an optimum strength of 5′ splice site, implicating variation in alternative splicing events in the NMII family (Fig. S1*C*ii-iii).

Despite its weak 5′ splice site, A2 exon expression is maintained in neuronal cells; how is this possible? Competitive binding of splicing repressor proteins such as hnRNPA1 can obstruct the interaction of U1 and U2 snRNPs with the 5′ splice site and branch point sequences, respectively, and result in exon skipping. Conversely, if the exon has an intrinsically weak 5′ splice donor site that is in low complementarity to the RRM of U1 snRNP, it can also inhibit the recognition of the exon (29, 30). In such cases, the splice factors with RRMs specifically targeting the 5′ splice site or other cis-regulatory motifs, such as RbFox3, can facilitate exon recognition and binding of the U1 snRNPs to the exon-intron boundary, thus resulting in exon inclusion (31). Our study supports earlier studies by Kawamoto *et al.*, 1996, which showed that alternative splicing of NMHC IIB1 was regulated by RbFox proteins (32, 33). Although their study demonstrated that the binding of RbFox3 to the intronic enhancer motif, UGCAUG, of NMHC IIB1 caused inclusion of B1 exon, we found no such consensus binding motifs across the introns and A2 exon. Therefore, we propose the possibility of a unique regulatory mechanism of alternative splicing of A2 exon by RbFox3, facilitated by the binding of RbFox3 to the GUGACU motif near the 5’splice site of A2 exon. Further studies are warranted to explore other putative binding regions of RbFox3 across the A2 exon (18, 34) or the brain-specific splice factors other than RbFox3 that may function independently or in association with RbFox3 to bind to the NMHC IIA pre-mRNA and modulate A2 inclusion in brain tissues. This is very likely, since RbFox isoforms frequently function in cooperation with other splice factors (35). RbFox2 binds cooperatively with MBNL-1 to regulate pluripotent stem cell differentiation, whereas RbFox1 functions competitively with MBNL-1 to regulate the splicing program in myotonic dystrophy (36, 37). The putative binding site of MBNL-1 on the A2 exon suggests possible cooperative binding with RbFox3 for A2 exon inclusion, which needs to be further explored.

Despite the low abundance of A2 transcript at the transcript level (9), we were able to detect an increased level of A2 splicing when both exogenous RbFox3 and 5′ splice site mutants of A2 exon (A2M1 or A2M2) are present in Neuro2A cells (Fig. 4, *C* and *D* and S3*A*). This suggests that both cis and trans-regulatory factors should be above the threshold value for abundant expression of the inactive NMIIA2 isoform. Understanding the tissue-specific splice factors that restrict the expression of these proteins in neuronal cells is important to correlate their expression with their roles in neuritogenesis, neurite branching, neuronal cell synapsis, cell adhesion and migration (38). Recently, Heo *et al.* (2025) demonstrated that NMII inhibition promotes axonal regeneration and functional recovery, indicating that the intrinsic activity of NMIIA may be locally repressed at the site of axonal injury by its enzymatically inactive isoform, NMIIA2, through heterofilament formation (39). Our previous study demonstrated that NMIIA2 can form heterofilaments with NMIIA and reduce the overall NMIIA activity (9).

Thus, the regulation of loop2 alternative splicing in NMHC IIA by RbFox3 can modulate the expression of its spliced isoforms, NMIIA0 and –IIA2, to generate NMIIs with various enzymatic properties. Further investigation is warranted to elucidate how the heterofilaments of the inserted and non-inserted isoforms regulate the endogenous activities of the non-inserted isoforms and their implications in neuronal cell development and neuritogenesis.

## Experimental procedures

### Cloning of the A2 exon containing genomic DNA fragment

Genomic DNA from mouse 4T1 cells was isolated and amplified by PCR using Q5 High Fidelity DNA polymerase (NEB, Massachusetts, United States, #M0491). The P1 and P4 primers (Table S2) were designed to amplify the genomic DNA fragment that covers the introns flanking exons 15 and 16 of the mouse *Myh9* gene to generate the PCR fragment of 3.8 kb (L). Another pair of primers, P2 and P3, was designed to amplify the genomic DNA fragment that covers the introns flanking only the A2 exon of *Myh9* gene to generate a PCR fragment of 1.8 kb (S). The two PCR products, 3.8 kb and 1.8 kb, were individually cloned into a TA vector (pCRII TOPO) to generate plasmids named as L-TOPO and S-TOPO, respectively, followed by subcloning at the SpeI/SacII site or ApaI/SacII site of pET01 Exontrap vector (MoBiTec GmbH), and named as L-pET01 and S-pET01, respectively.

Six mutant constructs were prepared from the wildtype L-pET01 plasmid. Site directed mutagenesis (SDM) was performed using Q5 Site-Directed Mutagenesis Kit (NEB, #0554) to mutate the 5′ splice site of A2 exon from GTGACT to GTGAGT (C>G) to generate A2M1-L-pET01 plasmid and from GTGACT to GTAAGT (G>A and C>G) to generate A2M2-L-pET01 plasmid. Moreover, the branch point (BP) sequence was mutated in the wildtype L-pET01 and A2M1 plasmid to generate two additional plasmids: BP3-L-pET01, containing only the mutation at the BP from CTCTCAT to CTCTAAC(C>A and T>C) and BP3A2M1-L-pET01, containing the mutation from CTCTCAT to CTCTAAC(C>A and T>C) and GTGACT to GTGAGT (C>G) at the BP and 5′ splice site of A2 exon, respectively. The intron sequence between exon 15 and A2 exon is known as the Left Intron Sequence Element (LIS) and the intron sequence between A2 exon and exon 16 is known as the Right Intron Sequence Element (RIS). The introns flanking A2 exon were truncated to generate ΔLIS and ΔRIS-pET01 deletion constructs. In ΔLIS-pET01 plasmid, the 214 nucleotides were deleted from LIS while keeping 100 nucleotides from the intron-exon boundary intact, using SDM. In ΔRIS-pET01 plasmid, the 992 nucleotides were deleted from RIS, retaining 200 nucleotides from each end of the intron, using ligation-based cloning. During RIS cloning, re-ligation of the truncated linearized DNA included a NotI linker to mark the joining position. Two mutant constructs were prepared from the wildtype S-pET01 plasmid using SDM. The 5′ splice site of A2 exon in S-pET01 plasmid was mutated from GTGACT to GTGAGT (C>G) to generate A2M1-S-pET01, and from GTGACT to GTAAGT (G>A and C>G) to generate A2M2-S-pET01 plasmids. All constructs were verified by digestion using multiple restriction enzymes and sequencing.

### Cell culture and plasmid DNA transfection

The mouse neuroblastoma, Neuro2A and breast cancer, 4T1 cell lines were obtained from American Type Culture Collection (ATCC), and maintained in growth medium composed of low glucose DMEM (Thermo Fisher, #11885092) and RPMI (Thermo Fisher, #11875093), respectively, supplemented with 10% fetal bovine serum (FBS, Thermo Fisher, #10082147) and 1% penicillin-streptomycin (Thermo Fisher, #10378016). All cell lines were maintained in a 37 °C incubator with 5% CO_2_, and were routinely tested for *mycoplasma* contamination in the laboratory. The cells were transfected with plasmid DNA using Lipofectamine 3000 (Invitrogen, #L3000001). Typically, 1-1.5 μg of plasmid DNAs were transfected to 2 × 10^6^cells in a 35 mm culture dish. The transfected cells were maintained in an incubator for 48 h before being harvested for total RNA isolation. In experiments involving sequential co-transfection of RbFox, MBNL-1 or NOVA-1 and wildtype or mutant pET01 plasmids, Neuro2A cells were first transfected with pCGT7-Nova-1, VENUS-C1-MBNL-1, pCMV-RbFox1-FLAG, pCMV-RbFox2-FLAG, or pCMV-RbFox3-FLAG. After 24 h, cells were transfected with wildtype or mutant S-pET01 or L-pET01 plasmids. The co-transfected cells were harvested at 48 h post-transfection for total RNA isolation.

### Isolation of total RNA and RT-PCR

Total RNA was isolated from transfected Neuro2A cells at 48 h post-transfection using TRIzol reagent (Invitrogen, #15596026) and the total RNA from three replicates was pooled and reverse transcribed using RT2 First Strand Kit (Qiagen, #330401) according to the manufacturer’s protocol. PCR was carried out with the indicated primer sets (Table S2) using ReadyMix Taq PCR Reaction Mix (Sigma-Aldrich, #P4600). All RT-PCR experiments included a no-RT control, which gave no PCR product. The markers used for the study included MassRuler Low Range DNA Ladder (Thermo Fisher, #SM0383).

### RNA immunoprecipitation

RNA immunoprecipitation was performed according to the previously published protocol (40). Briefly, Neuro2A cells were either transfected with wildtype S-pET01 alone or co-transfected with pCMV-RbFox3-FLAG. At 48 h post-transfection, crosslinking was performed using 0.5% paraformaldehyde for 10 min followed by quenching the crosslinking reaction with 125 mM Glycine for 5 min, at room temperature (r.t.). Ice-cold cell lysis buffer (50.0 mM Tris–HCl pH 7.5, 150.0 mM NaCl, 10.0 mM EDTA, 0.5% vol/vol NP-40, and 0.1% vol/vol Triton X-100, 0.1% Sodium deoxycholate, 1 mM PMSF, RNase inhibitor (Qiagen, #129916) and protease inhibitor (Sigma-Aldrich, #P8340)) was used to lyse the cells. The crosslinked ribonucleoproteins were solubilized by 10 rounds of water sonication, 10 s each, at 50% amplitude followed by removal of insoluble material by centrifugation at 15,000*g* for 10 min at 4C. The FLAG-tagged RbFox3 was immunoprecipitated by incubating the cell lysate with FLAG antibody (Sigma-Aldrich #F1804) for 6 h at 4C and IgG (Sigma-Aldrich) was kept as a negative control. Protein G agarose beads were added to the antibody-antigen mixture and kept overnight at 4C. The immunoprecipitated pellet was obtained by centrifugation at 3000 rpm for 3 min while the supernatant was collected separately. After washing the pellet three times with PBS buffer, elution was done using elution buffer (50.0 mM Tris–HCl pH7.5, 5 mM EDTA, 10 mM DTT and 1% SDS). The pellet was partially stored for immunoblot analysis of the FLAG immunoprecipitation and the rest was used for RNA isolation. Proteinase K (Thermo Fisher #EO0491)-pre-treated pellet was heated at 65C for 1 h with 10 mM NaCl for crosslinking reversal and Trizol was added for RNA isolation.

### Immunoblotting

Cell lysates of 2 x 10^6^Neuro2A cells were prepared in a lysis buffer composed of 50 mM Tris-HCl (pH 8.0), 60 mM KCl, 10 mM MgCl2, 5 mM ATP, 4 mM EDTA, 1 mM DTT, 1% Nonidet P-40, 0.5 mM PMSF, protease (Sigma-Aldrich #P8340) and phosphatase inhibitors cocktail (Sigma-Aldrich #P5726). The lysates were centrifuged at 12,000*g* for 15 min at 4 °C, and supernatant was fractionated by 6 to 8% SDS-PAGE and transferred onto 0.45 μm PVDF membranes. The membranes were blocked with 5% BSA (Sigma-Aldrich, #A7030) for 6 h at 4 °C followed by overnight incubation at 4 °C with primary antibodies specific to NMHC IIA2 (9), NMHCIIA (Sigma-Aldrich, #M8064), GAPDH (Sigma-Aldrich, #G8795) or FLAG (Sigma-Aldrich, #F1804). Then, the blots were incubated with HRP-conjugated anti-mouse (Sigma-Aldrich, #A8924) or anti-rabbit secondary antibody (Thermo Fisher, #32460) for 2 h at rt. Chemiluminescence signals were visualized using Super Signal Femto Reagent (Thermo Fisher, #34095) and captured using a Chemidoc Touch Imaging system (Bio-Rad). Quantification of band intensity was carried out using Fiji (Image J) software (NIH).

### Immunofluorescence, confocal microscopy, and image analysis

Neuro2A cells transfected with RbFox3-FLAG were fixed with 4% paraformaldehyde for 20 min, permeabilized with 0.5% Triton X-100 for 10 min and blocked with 3% BSA for 1 h at r.t. Cells were then incubated at 4 °C overnight with NMHC IIA2 and FLAG (Sigma-Aldrich, #F1804) antibodies. Secondary antibodies conjugated with Alexa Fluor 488 (Thermo Fisher, #A-11017) or 594 (Thermo Fisher, #A-11012) were added and incubated for 1 h. The nucleus was counterstained with DAPI (Sigma-Aldrich, #D8417). Samples were mounted using Prolong gold antifade reagent (Thermo Fisher, #P36934). Images were captured using a Carl Zeiss LSM 880 confocal microscope (Zeiss) and processed using Airyscan. To quantify the intensity of NMIIA2 and FLAG-tagged RbFox3 proteins in Neuro2A cells, two ROIs were considered: the ROI of the nucleus, I_nucleus_ (stained by DAPI) and the ROI of the cell, I_cell_, (bright-field image). The mean intensity of Alexa 488 was calculated as: I_cell_, and cytoplasmic NMIIA2 and was calculated as: I_cytoplasm_= [I_cell_ - I_nucleus_], and the line graph was plotted considering Alexa-488 intensity (Y-axis) *versus* NMIIA2 intensity (X-axis).

#### *In silico* analyses

The strength of 5′ splice donor site, 3′ splice acceptor site, the position and strength of BP sequences, Exonic Regulatory Sequences (Exon Splicing Enhancers, Exon Splicing Silencers, Intron Splicing Enhancers and Intron Splicing Silencers) and splice factors were determined using *in silico* splicing signal analysis tools, as previously published (41). Briefly, the input was the *Mus musculus* genomic DNA sequence consisting of exon 15, 16 and A2 and the flanking introns. The gene locus of the input sequence in the *Myh9* gene of: 77665808 to 77666089 (NC_000081.7: *M. musculus* GRCm39 Chr 15, Gene Myh9). MaxEntScan, was used to determine the scores of the 5′ and 3′ SS (11, 12, 41). BP sequences were screened using HSF 3.1 (Human Splicing Finder - Version 3.1 – UMD, Genomnis) (13, 30). SpliceAid 2 was used to detect the splicing signals in the Exon 15, 16 and A2 exons and the intermediate introns (16). The splicing signals included the splice factors and the sequence motifs to which these splice factors can bind (ESE, ESS, ISE and ISS). In SpliceAid2, the tissue type was specified as “Brain” as a filter parameter to limit the search to splice factors expressed in the brain. The intrinsic preset thresholds were used in each of these algorithms. RNAfold (ViennaRNA package v2.0) was used to generate the RNA secondary structures using the input sequence consisting of the A2 exon and flanking intronic sequences (1873 nt) and the minimum entropy structure was selected for representation (15). The input sequences for the wildtype, A2M1 and A2M2 were identical differing only at the position of mutated nucleotides and secondary structures were generated using the default parameters of the software. The *in silico* analyses of the human A2 exon sequence, which included the strength of 5′ and 3′ SS, RNA secondary structure and the RBP binding site predictions, were carried out using MaxEntScan, RNAfold and SpliceAid2, respectively, using identical input parameters as those used for the mouse A2 exon sequence.

## Statistical analysis

Statistical analysis and graphs were prepared using Origin 2019b software. Data are represented as mean ± SD of values obtained from three independent experiments. The Student’s *t* test was used to compare the means between control and experimental groups. The differences between the means of multiple groups were analyzed using two-way analysis of variance (ANOVA) with Tukey’s multiple comparison tests. ∗*p*< 0.05, ∗∗*p*< 0.01, ∗∗∗*p*< 0.001, were considered significant whereas *p* > 0.05 was considered non-significant (ns).

## Data availability

All the data presented in this study are available in the article and supplementary information.

## Supporting information

This article contains supporting information.

## Conflict of interest

The authors declare that there is no conflict of interest among the authors in the present work
