# Supplementary material for: Binding of RbFox proteins at weak 5’splice site of A2 induces its alternative splicing in non-muscle myosin heavy-chain IIA mRNA
Source: J Biol Chem. 2026 Apr 9;302(6):111443. doi: 10.1016/j.jbc.2026.111443 (PMC13158602; doi:10.1016/j.jbc.2026.111443)
Supplement: Supporting Information [file mmc1.pdf]

Supporting Information

Binding of RbFox proteins at weak 5'splice site of A2 induces its alternative splicing in Non-muscle Myosin Heavy Chain IIA mRNA

Ditipriya Mallick[1], Nitish Pal[1], Sampurna Dutta[1], Siddhartha Sankar Jana*[1]

[1]*Indian Association for the Cultivation of Science, Kolkata, India.*

*Address for Correspondence: bcssj@iacs.res.in

A.

NC_000077.7 Mus musculus GRCm39 Chr 11, *Myh10*

```
     Mouse tccactgtttctctgccttaGAGATTCAGACTATTCAGAGAGCTTCTTTCTATGACAGTGTTTCTGGTCTTCATGAGCCACCAggtgaatgtatacagcctgc 103
     Human tttgctgtttctctgctttaGAGATTCAGAATATTCAGAGAGCTTCTTTCTATGACAGTGTTTCTGGTCTTCATGAGCCACCAggtgaatgtataaagcctgt 103
       Rat ttcactgtttctctgccttaGAGATTCAGAATATTCAGAGAGCTTCTTTCTATGACAGTGTTTCTGGTCTTCATGAGCCGCCAggtgaatgtatacagcctgc 103
       Cow tttgctgtttctctgctttaGAGATTCAGAATATTCAGAGAGCTTCTTTCTATGACAGTGTTTCTGGTCTTCATGAGCCACCAggtgaatggataaagcctgt 103
       Pig tttgctgtttctctgctttaGAGATTCAGAATATTCAGAGAGCTTCTTTCTATGACAGTGTTTCTGGTCTTCATGAGCCACCAggtgaatggataaagcctgt 103
 Alligator tttgctgtttctctgccttaGAGATTCAGAATATTCAGAGAGCCTCTTTCTATGACAATATTACTGGTCTTCATGATGCACCAggtgaatgtatacaaaaact 103
Chimpanzee tttgctgtttctctgctttaGAGATTCAGAATATTCAGAGAGCTTCTTTCTATGACAGTGTTTCTGGTCTTCATGAGCCACCAggtgaatgtataaagcctgt 103
     Otter tttgctgtgtctctgctgtaGAGATTCAGAATATTCAGAGAGCTTCTTTCTATGACAGTGTTTCTGGTCTTCATGAGCCACCAggtgaatgtacaaagcctgt 103
     Camel tttgctgtttctctgctttaGAGATTCAGAATATTCAGAGAGCTTCTTTCTATGACAGTGTTTCTGGTCTTCATGAGCCACCAggtgaatgtataaagcctgt 103
   Chicken tttgctgtttctctgccttaGAGATTCAGAATATTCAGAGAGCCTGTTTCTATGACAATATTACTGGTCTTCATGATCCACCAggtgaatggatgtaaaacca 103
                                ************ ************ * *********** * ** *************  * *****
```

B.

NC_000073.7 Mus musculus GRCm39 Chr 7, *Myh14*

```
     Mouse ccccagAACAAGGGGGTCTCCAACAGTTCACTTTACTTGGCTCCTTCCCATCGCCGTCCCCTGGACCTGCAGGGAGACTCGGCTCTGGCGCTTCTCCTCCAGGGGTTGGGTCTCTCTGTGCACCCACGGgtgagg   135
     Human ccccagAACATGGGGGCTTCCAGCAGTTCTCTTTCCTTGGCTCCTTCCCACCGTCGCCCCCAGGATCTGCAGAGAGGTGCAGCTCTGCTATTTCTCCGCCAGGGGGTGGGTGTCTCTGTGCATCGATGGgtgagg   135
       Rat ccccagAACAAGGGGGTCTCCAACAGTTCACTTTCCTTGGCTCCTTCCCGTCGCCGTCCCCTGGACCTGCAGGGGGACTCAGCTCTGGTGCTTCTCCTCCAGGGCTTGGGTCTCTCTGTGCACCCACGGgtgagg   135
       Cow ccccagAACACGGGGGCTTGCAGCAGTTCTCTTTCCTTGGCTCCTTCCCACCGTCGCCCCCAGGATCTTCAGGGAGGCACAGCTCTGCCATTTCTCCATCAGGGGGTGGGTGTTGCTGTGCATCAACGGgtgaga   135
       Pig ccccagAACATGGGGGCTTACAACCGTTCTCTTTCCTTGGCTCCTTCCCACCGTCGCCCCCAGGATCTTCAGGGAGGCGCGGCTCCGCCACTTCTCCGTCAGGGGGTGGGTGTTGCTGTGCATCAATGGgtgaga   135
Chimpanzee ccccagAACATGGGGGCTTCCAGCAGTTCTCTTTCCTTGGCTCCTTCCCACCGTCGCCCCCAGGATCTGCAGAGAGGTGCAGCTCTGCTAGTTCTCCGCCAGGGGGTGGGTGTCTTTGTGCATCGATGGgtgagg   135
   Hamster ccccag-AACACAGGGCCTCCACCAGTTCACTTCACTTGACTTGTTCCCATCG-TCACCCCTGGACCTGCAGGGACACTCAGCTCTGGGGCTTCCCCGCCAGGGGGTGGGTCTCTCTGTGCACCCACGGgtgaga   135
   Macaque ccccagAACACGGGGGTTTCCAGCAGTTCTCTTTCCTTGGCTCCTTCCCACCGTCGCCCCCAGGATCTGCAGAGAGGTGCAGCTCTGCTGTTTCTCCGCCAGGGGGTGGGTGTCTCTGTGCATCAATGGgtgagg   135
   Gorilla ccccagAACATGGGGGCTTCCAGCAGTTCTCTTTCCTTGGCTTCTTCCCACCGTCGCCCCCAGGATCTGCAGAGAGGTGCAGCTCTGCTATTTCTCCGCCAGGGGGTGGGTGTCTTTGTGCATCGATGGgtgagg   135
     Camel acccagAACACGGGGGCTTGCAGCAGTTCTCTTTCCTGGGCTCCTTCCCACCGTCGCCCCCAGGATCTCCAGGGAGGCACAGCTCTGCCATTTCTCCGTCAGGGGGTGGGTGTTGCTGTGCACCAACGGgtgaga   135
                ** *     ***  * ** * **** ***  ** * **  *****  **    **** *** ** *** *      * **** *    *** **  *****  ***** *   ****** * * ****
```

C.

(i)

3' Acceptor	Max. Ent. Score	Threshold
Exon 15	11.83	7.02
A2 Exon	9.02	7.46
Exon 16	4.03	4.01

(ii)

3' Acceptor	Max. Ent. Score	Threshold
Exon 16	1.664	1.0
B2 Exon	6.78	2.2
Exon 17	10.82	2.2

5' Donor	Max. Ent. Score	Threshold
Exon 16	10.002	4.5
B2 Exon	8.77	4.5
Exon 17	7.03	4.5

(iii)

3' Acceptor	Max. Ent. Score	Threshold
Exon 14	9.42	6.72
C2 Exon	8.37	6.68
Exon 15	7.97	6.72

5' Donor	Max. Ent. Score	Threshold
Exon 14	4.59	4.5
C2 Exon	9.48	6.72
Exon 15	9.25	6.68

D.

(i)

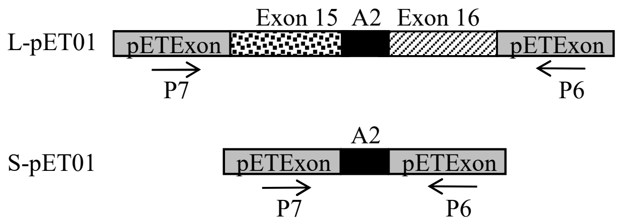

(ii)

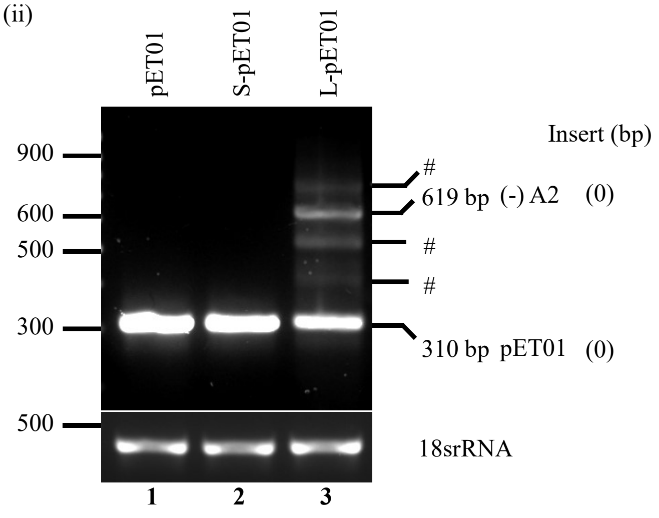

Figure S1

Fig. S1. Alternative splicing of A2 exon in S-pET01 and L-pET01 and analysis of its cis-regulatory sequences.

A-B. The genomic location of mouse NMHC IIB and -IIC gene, corresponding to the region consisting of B2 and C2 exon (uppercase letters), respectively, and the flanking introns (lowercase letters). The boldface, underlined letters denote the 3' splice site and the boldface, italics and underlined letters denote the 5' splice site, of the A2 exon. Sequence alignment of the genomic region across the 10 species. Asterisks (*) indicate sequence identity and boldface letters denote non-identity at alternative exon. **C.** The MaxEntScan score of the 3' splice site of the A2 exon and the flanking exons (i), 3' and 5' splice sites of the B2 (ii) and C2 (iii) exons and the flanking exons. **D.** A schematic map of the exon arrangement as generated from S-pET01 and L-pET01, arrows indicate the positions of the primers used for RT-PCR (i). RT-PCR analysis using pET01 exon-specific primers showing spliced products generated from S-pET01 and L-pET01 transfected in Neuro2A cells (ii). (#) denotes other spliced products not corresponding to pET01, (-) or (+) A2 exon.

A.

(i)

Donor	Sequence	Max. Ent. Score
Wildtype	GTGACT	6.60
A2M1	GTGAGT	10.67
A2M2	GTAAGT	10.86

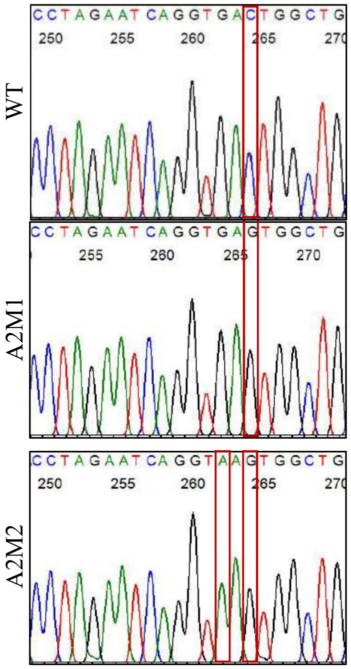

(ii)

Distance from A2 exon	Branch - point motifs in LISE	Consensus value (0-100)
-107	gcctctc	68.51
-105	ctctctc	66.77
-95	tcctcag	95.51
-56	gcctctc	68.51
-54 (WT)	ctctcat	92.5
-43	ccctctc	70.14
-38	tcctccg	65.88
-15	acctctc	65.89
-5	tgcagag	70.91
Distance from A2 exon	Branch - point motifs in LISE	Consensus value (0-100)
-54 (BP3)	ctctaac	84.57

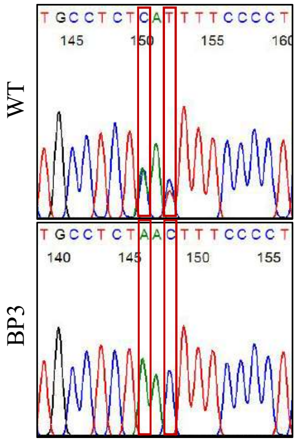

B.

Human

5' Donor	Max. Ent. Score	Threshold
Exon 15	8.57	6.678
A2 Exon	6.60	6.5
Exon 16	8.17	7.14

3' Acceptor	Max. Ent. Score	Threshold
Exon 15	8.89	7.02
A2 Exon	9.02	7.46
Exon 16	8.79	4.01

C.

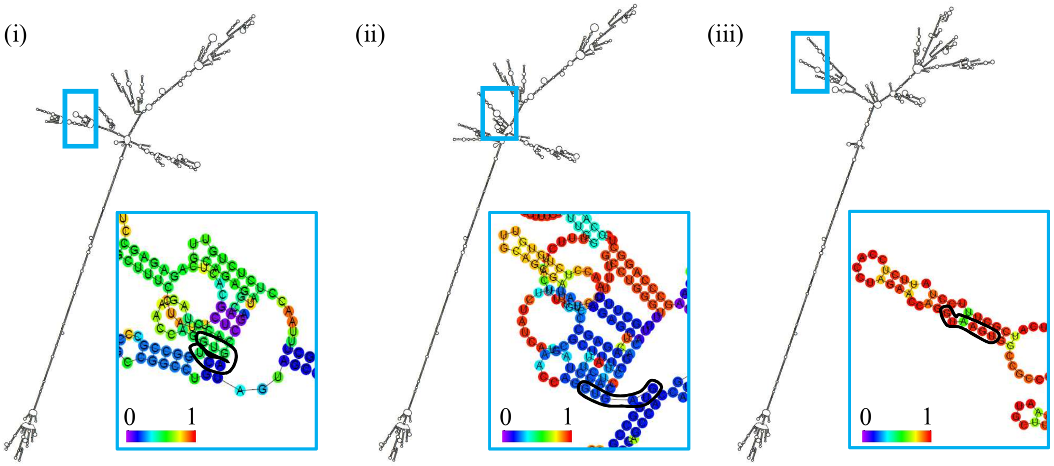

WT (Human)
ΔG: **-1096.40** kcal/mol

A2M1 (Human)
ΔG: **-1098.30** kcal/mol

A2M2 (Human)
ΔG: **-1097.10** kcal/mol

D.

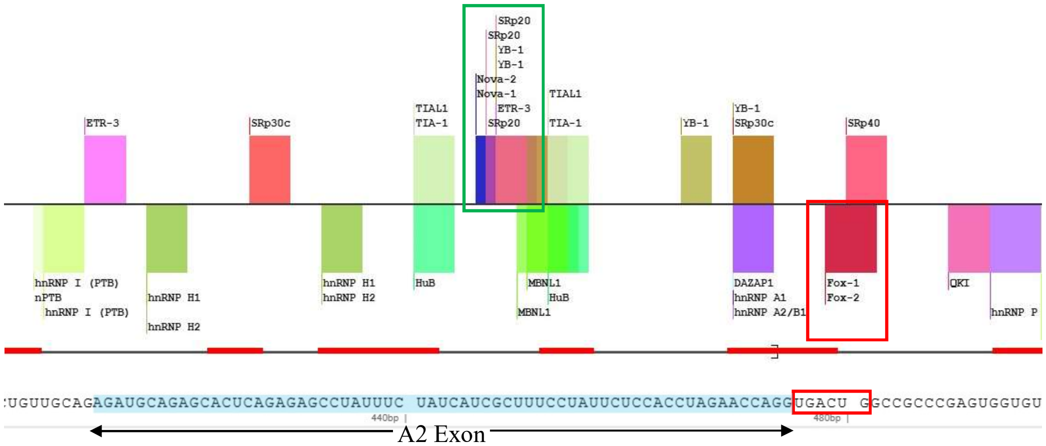

Figure S2

Fig. S2. Increasing the strength of 5’ splice donor site causes A2 exon inclusion.

A. The MaxEntScan score of the 5’ splice site (left) and the chromatograms (right) corresponding to the A2 exon (wildtype, highlighted) or mutants (A2M1 and A2M2) (i). The putative branch-point sequences in the LIS region of A2 exon, as predicted by HSF3.0 (ii). The highlighted branch-point sequence closest to the consensus value and within 60bp from the A2 exon was mutated in BP3-L-pET01 or BP3-A2M1-L-pET01, as shown in the chromatogram (right). **B.** The MaxEntScan score of the 3’ and 5’ splice sites of the human A2 exon and the flanking exons. **C.** RNA secondary structures generated using RNAfold webserver of the region corresponding to the human A2 exon and flanking introns in WT **(i)** or MT (A2M1 and A2M2) **(ii-iii)**. The boxed region comprising of the stem-loop structure of A2 exon is enlarged (below) to show the base pair probabilities of the nucleotides in the RNA model, where the nucleotides at 5’ splice sites of WT and MT are outlined in black. **D.** SpliceAid2 generated putative neuronal splice factor binding sites on the human A2 exon and the flanking introns. The additional binding of splice factors on the human A2 sequence as compared to mice A2 sequence is boxed in green, while the RbFox binding site and 5’ splice site are boxed in red.

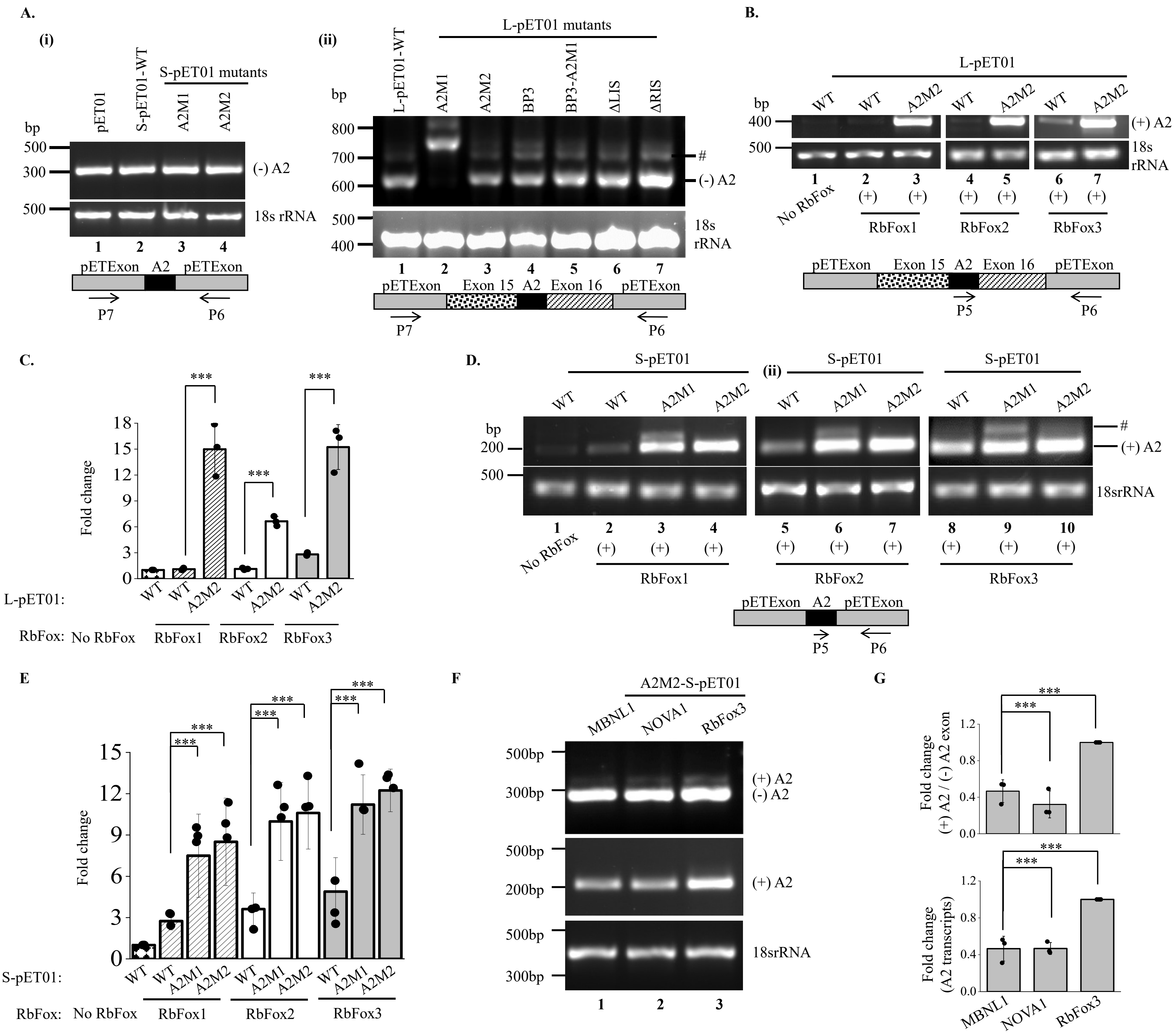
A.
(i)
pET01
S-pET01-WT
S-pET01 mutants
A2M1
A2M2
bp
500
300
500
(-) A2
18s rRNA
1 2 3 4
pETExon A2 pETExon
P7
P6
(ii)
L-pET01-WT
L-pET01 mutants
A2M1
A2M2
BP3
BP3-A2M1
ΔLIS
ΔRIS
bp
800
700
600
500
400
#
(-) A2
18s rRNA
1 2 3 4 5 6 7
pETExon Exon 15 A2 Exon 16 pETExon
P7
P6
B.
L-pET01
WT WT A2M2 WT A2M2 WT A2M2
bp
400
500
(+) A2
18s rRNA
1 2 3 4 5 6 7
No RbFox
(+) (+) (+) (+) (+) (+)
RbFox1
RbFox2
RbFox3
pETExon Exon 15 A2 Exon 16 pETExon
P5
P6
C.
Fold change
0 3 6 9 12 15 18
***
***
***
L-pET01:
WT WT A2M2 WT A2M2 WT A2M2
RbFox: No RbFox RbFox1 RbFox2 RbFox3
D.
S-pET01
(ii)
S-pET01
S-pET01
WT WT A2M1 A2M2 WT A2M1 A2M2 WT A2M1 A2M2
bp
200
500
#
(+) A2
18srRNA
1 2 3 4 5 6 7 8 9 10
No RbFox
(+) (+) (+) (+) (+) (+) (+) (+) (+)
RbFox1
RbFox2
RbFox3
pETExon A2 pETExon
P5
P6
E
Fold change
0 3 6 9 12 15
***
S-pET01:
WT WT A2M1 A2M2 WT A2M1 A2M2 WT A2M1 A2M2
RbFox: No RbFox RbFox1 RbFox2 RbFox3
F
A2M2-S-pET01
MBNL1
NOVA1
RbFox3
500bp
300bp
200bp
(+) A2
(-) A2
18srRNA
1 2 3
G
Fold change
(+) A2 / (-) A2 exon
(A2 transcripts)
0.0 0.4 0.8 1.2
***
MBNL1
NOVA1
RbFox3

Figure S3

Fig. S3. RbFox proteins bind to the 5' splice site to promote A2 exon inclusion in transcripts generated from S-pET01 and L-pET01 minigene.

A. RT-PCR analysis using pET01 exon-specific primers showing spliced product generated from S-pET01 wildtype or mutants transfected in Neuro2A cells. A schematic map of the exon arrangement generated from S-pET01, arrows indicate the positions of the primers used for RT-PCR (below) (i). RT-PCR analysis using pET01 exon-specific primers showing spliced product generated from L-pET01 wildtype or mutants transfected in Neuro2A cells. (#) denotes other spliced products not corresponding to pET01, (-) or (+) A2 exon. A schematic map of the exon arrangement generated from L-pET01, arrows indicate the positions of the primers used for RT-PCR (below) (ii). **B.** RT-PCR analysis using A2 exon-specific primer showing spliced products generated from L-pET01 wildtype or mutant (A2M2) co-transfected with (+) or without (-) RbFox1, RbFox2 or RbFox3-FLAG, in Neuro2A cells. 18s rRNA was used as a loading control. A schematic map of the exon arrangement generated from L-pET01, arrows indicate the positions of the primers used for RT-PCR (below). **C.** Fold change of expression of A2 exon-included transcripts. **D.**RT-PCR analysis using A2 exon-specific primer showing spliced products generated from S-pET01 wildtype or mutants (A2M1 or A2M2) co-transfected with (+) or without (-) RbFox1, RbFox2 or RbFox3-FLAG, in Neuro2A cells. 18s rRNA was used as a loading control. A schematic map of the exon arrangement generated from S-pET01, arrows indicate the positions of the primers used for RT-PCR. (#) denotes uncharacterized spliced product in A2M1-S-pET01. Please note that 18s rRNA RT-PCR shown in Figure S3D has been reused from the first 18s rRNA panel of Figure 4C(i) as all the panels of both figures derive from the same experiment in which identical samples have been analyzed by RT-PCR using different sets of primer as mentioned below the panels. **E.** Fold change of expression of A2 exon-included transcripts. **F.** RT-PCR analysis using pET01 exon-specific primer or A2 exon-specific primers, showing spliced products generated from A2M2-S-pET01 co-transfected with RbFox3, NOVA1 or MBNL1 in Neuro2A cells. 18s rRNA was used as a loading control. **G.** Fold change of expression of A2 exon-included (+A2) /A2 exon-excluded transcripts (-A2) and A2 exon-included transcripts. ***, $p<0.001$; WT (+) vs A2M1 (+) or A2M2 (+); RbFox3 vs MBNL1 or NOVA1.

Table S2.

Primer	Sense	Sequence 5'-3'
**Cloning Primers**		
P1 (L-pET01)	Forward	GTCGACGGGCCCGTGAGGCGGCCAGAGAAGGGT
P4 (L-pET01)	Reverse	GCGGCCGCCCGCGGCTGGAGAGGAAAGCACATGGA
P2 (S-pET01)	Forward	GTCGACGGGCCCGTACGCCTGCCCTTTGCCCTC
P3 (S-pET01)	Reverse	GCGGCCGCCCGCGGCTGTGAAGAACATATAGCTTG
A2M1	Forward	GAATCAGGTGAGTGGCTGCCTGC
A2M1	Reverse	TAGGTGGAGAATGGGAAAGCG
A2M2	Forward	CTAGAATCAGGTAAGTGGCTGCCTGC
A2M2	Reverse	GTGGAGAATGGGAAAGCGG
BP3	Forward	TTTCTGCCTCTAACTTTCCCCTCTCC
BP3	Reverse	AAGAACAGCTTCAAGGCAGC
ΔLIS	Forward	GCGGCCGCTGCCTCTCTCTCCTCCTCA
ΔLIS	Reverse	GCGGCCGCCCAGTGTCATGGTCCTTTGT
ΔRIS	Forward	GCGGCCGCCTCTGTGCTTTGCTGTCGTC
ΔRIS	Reverse	GCGGCCGCCCCAGTCCTTCAGAAGCATG
**PCR Primers**		
P5 (A2 Exon)	Forward	AGCACTCAGAGAGCCTATTTCT
P8 (A2 Exon)	Reverse	TTGGTATTCCTCAACGTGGC
P7 (pET Exon)	Forward	TGGCCCTGCTCATCCTCTG
P6 (pET Exon)	Reverse	GTAGTGGTGGGCCTAGTTGCA
P9 (A0 Specific)	Forward	TGGAAGGATGTGGATCGGATCA
P10 (A0 Specific)	Reverse	TGCAGCGCACGAAGTTGGGG
18srRNA	Forward	CGGCTACCACATCCAAGGAA
18srRNA	Reverse	TCCATTATTCCTAGCTGCGG
pET01 overlap	Forward	ACCCACAAGGTAGTGGCACAAC
pET01 overlap	Reverse	TATTCATTGCAGAGGGGTGG

Table S2. List of Primers used in this study.
