# Supplementary material for: Binding of RbFox proteins at weak 5’splice site of A2 induces its alternative splicing in non-muscle myosin heavy-chain IIA mRNA
Source: J Biol Chem. 2026 Apr 9;302(6):111443. doi: 10.1016/j.jbc.2026.111443 (PMC13158602; doi:10.1016/j.jbc.2026.111443)
Supplement: Table S1 [file mmc2.pdf]

**Neuron-specific splice factors**	**Number and Sequence of Binding Site**	**Region of Binding**
nPTB	[4], CUCUCU	LISE
HuB	[1], CUUUC	LISE
PTB	[14], UCUUU	LISE
SF2/ASF	[4], CCCAUGA	LISE
MBNL-1	[1], CCGCUU	**A2 EXON**
TIA 1, TIAL-1	[2], AUUUC, [2] CUUUC	A2 EXON
HuB	[1], CUUUC	A2 EXON
**RBFOX1**	[1], UGCAUG, UGACUG	**5'SS, RISE**
**RBFOX2**	[1], UGCAUG, UGACUG	**5'SS, RISE**
**RBFOX3**	UGCAUG	**5'SS, RISE**
NOVA1	[10], YCAY Cluster	**RISE**
NOVA2	[5], YCAY Cluster	RISE
nPTB	[1], CUCUCU	RISE
PTB	[16], UCUUU	RISE
HuB	[3], CUUUC	RISE
Tra2 beta	[2], GAAGGA	RISE
ETR-3	[52], GUUGUU	RISE
SF2/ASF	[4], CCCAUGA	RISE

Table S1. The number and sequence of the binding sites and the binding region of the neuron-specific splice factors across the A2 exon, LISE and RISE, as determined by SpliceAid2.
